# Sgs1 Binding to Rad51 Stimulates Homology-Directed DNA Repair in *Saccharomyces cerevisiae*

**DOI:** 10.1534/genetics.117.300545

**Published:** 2017-11-21

**Authors:** Lillian Campos-Doerfler, Salahuddin Syed, Kristina H. Schmidt

**Affiliations:** * Department of Cell Biology, Microbiology and Molecular Biology, University of South Florida, Tampa, Florida 33620; † Graduate Program in Cell and Molecular Biology, University of South Florida, Tampa, Florida 33620; ‡ Cancer Biology and Evolution Program, H. Lee Moffitt Cancer Center and Research Institute, Tampa, Florida 33612

**Keywords:** Sgs1, Rad51, homologous recombination, DNA damage, DNA repair

## Abstract

Accurate repair of DNA breaks is essential to maintain genome integrity and cellular fitness. Sgs1, the sole member of the RecQ family of DNA helicases in *Saccharomyces cerevisiae*, is important for both early and late stages of homology-dependent repair. Its large number of physical and genetic interactions with DNA recombination, repair, and replication factors has established Sgs1 as a key player in the maintenance of genome integrity. To determine the significance of Sgs1 binding to the strand-exchange factor Rad51, we have identified a single amino acid change at the C-terminal of the helicase core of Sgs1 that disrupts Rad51 binding. In contrast to an *SGS1* deletion or a helicase-defective *sgs1* allele, this new separation-of-function allele, *sgs1-FD*, does not cause DNA damage hypersensitivity or genome instability, but exhibits negative and positive genetic interactions with *sae2*Δ, *mre11*Δ, *exo1*Δ, *srs2*Δ, *rrm3*Δ, and *pol32*Δ that are distinct from those of known *sgs1* mutants. Our findings suggest that the Sgs1-Rad51 interaction stimulates homologous recombination (HR). However, unlike *sgs1* mutations, which impair the resection of DNA double-strand ends, negative genetic interactions of the *sgs1-FD* allele are not suppressed by *YKU70* deletion. We propose that the Sgs1-Rad51 interaction stimulates HR by facilitating the formation of the presynaptic Rad51 filament, possibly by Sgs1 competing with single-stranded DNA for replication protein A binding during resection.

DNA double-strand breaks (DSBs) can be induced exogenously by DNA-damaging agents, or form endogenously if the replisome collapses at a nick in the template strand or encounters a physical barrier that blocks progression of the replisome, such as a bound protein, DNA adduct, interstrand cross-link, or unusual DNA structure. Cells can repair such DSBs by homologous recombination (HR) or nonhomologous end-joining (NHEJ). In the event of a DSB, the NHEJ proteins Ku70/Ku80 (Ku) and HR proteins Mre11-Rad50-Xrs2 (MRX) initiate the repair process by binding to the DSB ends (Mimitou and Symington 2010). NHEJ is preferred in G1 as there is no sister chromatid, whereas HR is preferred during S phase and G2 (Ira *et al.* 2004; Barlow *et al.* 2008; Huertas *et al.* 2008). In S phase, Ku and MRX bind to the DSB first and recruitment of Sae2 activates short-range resection, removing Ku and MRX, and leaving a small 3′ single-stranded DNA (ssDNA) overhang (Trujillo *et al.* 2003; Mimitou and Symington 2008, 2010; Nicolette *et al.* 2010; Niu *et al.* 2010; Cannavo and Cejka 2014). These trimmed DNA ends are then more extensively resected by the 5′–3′ exonuclease Exo1 or by Dna2 nuclease (Mimitou and Symington 2008; Zhu *et al.* 2008; Levikova *et al.* 2017). Exo1 and Dna2 differ in that Exo1 can degrade the 5′ strand in double-strand DNA (dsDNA), whereas Dna2 requires the Sgs1 helicase to unwind the dsDNA to provide ssDNA on which Dna2 can act (Cannavo *et al.* 2013; Levikova *et al.* 2017). Long-range resection by Sgs1/Dna2 and Exo1 is redundant, with loss of both activities resulting in a severe resection defect and mutagenic repair (Mimitou and Symington 2008; Zhu *et al.* 2008; Doerfler *et al.* 2011). Sgs1 also interacts with Mre11, and this may help to recruit Sgs1/Dna2 to the DSB after initial resection (Lisby *et al.* 2004; Chiolo *et al.* 2005).

Rad52, an essential HR factor in budding yeast, then allows a recombinogenic Rad51 filament to assemble on the replication protein A (RPA)-coated 3′-overhang (New *et al.* 1998; Song and Sung 2000). Regulation of HR at this stage relies on the antirecombinase Srs2, which can disassemble Rad51 filaments (Krejci *et al.* 2003; Veaute *et al.* 2003; Liu *et al.* 2011). If the Rad51-mediated homology search is successful, the 3′ end of the invading strand is extended by DNA synthesis. In classic DSB repair, the second end of the DNA break is also captured to form a double Holliday Junction (dHJ), which can either be resolved by endonucleases Mus81/Mms4 or Yen1 to produce both crossovers and noncrossovers, or the HJs are converged and decatenated by the Sgs1/Top3/Rmi1 complex, resulting in only noncrossovers (Wu and Hickson 2003; Cejka *et al.* 2010b, 2012).

Sgs1 is a member of the highly conserved family of RecQ-like DNA helicases, which interact with a large number of proteins with functions in genome maintenance. Sgs1 not only interacts with Dna2, Mre11, and Top3/Rmi1, but also contains acidic regions in its long, unstructured N-terminal tail that are required for binding the ssDNA-binding protein RPA (Gangloff *et al.* 1994; Mullen *et al.* 2005; Ui *et al.* 2005; Hegnauer *et al.* 2012; Kennedy *et al.* 2013). Rad53 kinase, Top2 topoisomerase, and the nucleotide excision repair factor Rad16 have also been shown to physically interact with the N-terminal tail of Sgs1, whereas Rad51 and Mlh1 binding has been narrowed down to the region C-terminal to the helicase core (Watt *et al.* 1995; Fricke *et al.* 2001; Saffi *et al.* 2001; Wu *et al.* 2001; Dherin *et al.* 2009; Hegnauer *et al.* 2012).

Lack of Sgs1 results in increased sensitivity to DNA-damaging agents, shortened life span, missegregated chromosomes, and moderate accumulation of gross chromosomal rearrangements (GCRs), including characteristic recurrent translocations between short homologous, but nonallelic, sequences (Sinclair *et al.* 1997; Mullen *et al.* 2000; Myung *et al.* 2001; Fricke and Brill 2003; Schmidt *et al.* 2010a). Cells lacking Sgs1 exhibit growth defects or die in the absence of structure-specific endonucleases Mus81/Mms4 and Slx1/4, which resolve recombination intermediates and stalled replication forks, the HR factors MRX or Sae2, the antirecombinase Srs2, or the Rrm3 helicase, which regulates replisome progression (Lee *et al.* 1999; Mullen *et al.* 2001; Fricke and Brill 2003; Schmidt and Kolodner 2004; Torres *et al.* 2004; Pan *et al.* 2006; Syed *et al.* 2016).

This multitude of physical and genetic interactions has established Sgs1 as a key player in the maintenance of genome integrity. The molecular basis and functional significance of some of the physical interactions for HR are increasingly well understood, especially the interaction of Sgs1 with Top3/Rmi1 in dHJ dissolution, with Dna2 in DSB resection, and with mismatch repair factors in the suppression of mitotic and meiotic homeologous recombination (Mullen *et al.* 2000; Wang and Kung 2002; Spell and Jinks-Robertson 2004; Amin *et al.* 2010; Kennedy *et al.* 2013; Levikova *et al.* 2017). Here, to understand the role of the physical interaction of Sgs1 with Rad51 in homology-dependent DNA repair, we set out to identify a separation-of-function allele of *SGS1* that disrupts Sgs1-Rad51 binding and to characterize the genetic interactions of this *sgs1* allele in cells with replication-dependent DNA lesions.

## Materials and Methods

### Yeast strains and media

Yeast strains were derived from S288C strain KHSY802 (*MAT****a***, *ura3-52*, *trp1*Δ*63*, *his3*Δ*200*, *leu2*Δ*1*, *lys2-Bgl*, *hom3-10*, *ade2*Δ*1*, *ade8*, *hxt13*:: *URA3*). *SGS1* mutant alleles for amino acid changes K706A (*sgs1-HD*, pKHS787) and F1192D (*sgs1-FD*, pKHS786) were generated by site-directed mutagenesis (QuikChange, Stratagene, La Jolla, CA) of wild-type *SGS1* in pKHS360 (*pRS405-SGS1.TRP1*), and integrated in the chromosome under the control of the endogenous *SGS1* promoter by LiAc-mediated transformation, as previously described (Gietz and Woods 2006). Haploid strains with multiple mutant alleles were obtained by sporulating diploids heterozygous for the desired mutations and genotyping spores on selective media or PCR. All yeast strains used in this study are listed in Supplemental Material, Table S1. Yeast were grown at 30° in yeast extract, peptone, and dextrose (YPD) or synthetic complete (SC) media, as previously described (Mirzaei *et al.* 2011). Solid media was supplemented with 20 g/liter agar (US Biological).

### DNA damage sensitivity assays

The sensitivity of yeast cells in the exponential growth phase to HU and MMS was tested by spot assays, as previously described (Mirzaei and Schmidt 2012). Briefly, cell cultures were grown in liquid YPD medium to OD_600_ = 0.5, and 10-fold serial dilutions were spotted on YPD containing the indicated concentration of HU (US Biological) or MMS (Sigma [Sigma Chemical], St. Louis, MO). Images of colony growth were acquired every 24 hr for 5 days of incubation at 30° with a Gel-Doc IT Imaging system (UVP, San Gabriel, CA).

### GCR assay

Cells with GCRs were identified by simultaneous inactivation of *CAN1* and *URA3* on chromosome V, indicated by resistance to canavanine and 5-FOA (Can^r^ 5-FOA^r^). Cultures were grown for 2 days in ≥ 10 ml of YPD media. Viable cell counts were determined by plating dilutions on YPD agar plates, and cells with GCRs were identified by plating 0.25–15 ml on synthetic media lacking arginine and uracil and supplemented with 60 mg/liter canavanine (Sigma) and 1 g/liter 5-FOA (US Biological). The rate of accumulating GCRs was calculated as previously described (Schmidt *et al.* 2010b).

### Mutator assays and mutation spectrum analysis

Rates of accumulating mutations at the *CAN1* locus, or reversion mutations in the *hom3-10* or *lys2-Bgl* alleles, were determined by fluctuation analysis by the method of the median (Lea and Coulson 1949) in at least 14 cultures from at least two different isolates, as previously described (Reenan and Kolodner 1992). Cultures were grown overnight in 3–6 ml of YPD media. Viable cell counts were determined by plating dilutions on YPD agar plates, and 250 μl–6 ml were plated on synthetic media lacking arginine and supplemented with 60 μg/ml canavanine for selection of inactivation of *CAN1*, or on SC media lacking threonine or lysine to select for threonine (*hom3-10*) or lysine (*lys2-Bgl*) revertants, respectively. Median rates are reported with 95% C.I.s (Nair 1940). The spectrum of inactivating mutations at the *CAN1* locus was determined by sequence analysis of *CAN1* from canavanine-resistant colonies using primer pairs that anneal 50-bp upstream and 43-bp downstream of the *CAN1* ORF.

### Gap repair assay

Crossover and noncrossover outcomes in a gap repair assay were determined, as previously described (Mitchel *et al.* 2010). The *can1*::*his3*Δ*3′* allele from plasmid pSR800 was inserted at the chromosomal *CAN1* locus of wild-type, *sgs1*∆, and *sgs1-FD* cells. Cells were then transformed with linearized pSR987, which contains the *his3* template for gap repair and a counterselectable *URA3* marker. Plasmids pSR800 and pSR987 were kindly provided by Sue Jinks-Robertson (Duke University). His^+^ colonies were selected on SC media lacking histidine (SC-His). Whether His^+^ colonies had formed by crossover or noncrossover events was determined by their ability to grow on agar plates containing 5-FOA, indicating loss of *URA3*. Briefly, colonies were first grown in the absence of histidine and then in nonselective media (either liquid YPD or as patches on YPD agar), before being spotted or replica-plated on agar plates containing 1 g/liter 5-FOA. Fully grown spots/patches were scored as noncrossovers and those with few or no colonies were scored as crossovers. Over 140 His^+^ colonies for each strain from two independent *can1*::*his3-0*,Δ*3′* isolates of the wild-type strain, and *sgs1∆* and *sgs1-FD* mutants, were analyzed.

### Tetrad analysis

Diploid strains for tetrad dissection were derived from S288C strains provided by Richard Kolodner (University of California, San Diego) and are listed in Table S1. To generate the diploid KHSY4810—heterozygous for *sgs1*Δ, *rad52*Δ, and *exo1*Δ mutations—RDKY5290 was crossed to KHSY4805 (an *exo1*Δ *rad52*Δ spore obtained from a cross between RDKY2614 and RDKY2710). *RAD59* and *RAD51* deletions were obtained by HR-mediated integration of a selectable marker at these loci in RDKY2666 using the LiAc method (Gietz and Woods 2006), and diploids heterozygous for *sgs1*Δ, *exo1*Δ, and *rad51*Δ or *rad59*Δ, were obtained by crossing as described above. For tetrad dissections, diploids were grown overnight in YPD at 30° and starved of nitrogen in 0.1% potassium acetate. Asci were briefly incubated with zymolase and dissected on YPD agar plates using a micromanipulator mounted on an Axioscop 40 (Zeiss [Carl Zeiss], Thornwood, NY). YPD plates were incubated for 4 days at 30°, and spore germination and colony growth were documented at 24-hr intervals with a CCD camera mounted on a GelDoc-IT Imager (UVP).

### Pull-down assay and western blotting

Plasmid pKHS657, expressing GST-Sgs1^647–1447^, was created by inserting the last 2400 bp of *SGS1* into pGEX-6p-2 (GE Healthcare) using *Bam*HI and *Xho*I restriction sites. Stop codons and *F1192A* and *F1192D* mutations were introduced at the indicated positions by site-directed mutagenesis (Quikchange, Agilent Genomics). Sgs1 fragments were expressed in *Escherichia coli* BL21 (DE) in LB media (10 g/liter tryptone, 5 g/liter NaCl, and 5 g/liter yeast extract) supplemented with 1.5 μg/ml ampicillin for 3 hr in the presence of 1 mM IPTG. Cells were resuspended in 100 µl GST buffer (125 mM Tris and 150 mM NaCl, pH 8.0) plus Halt protease inhibitor cocktail (Pierce Chemical, Rockford, IL), lysed using glass beads with a BeadBeater (Biospec Products) at 4°, and lysate cleared by centrifugation at 14,000 rpm for 10 min at 4°. Lysate was treated with benzonase (Sigma), and 1 mg of lysate was added to glutathione magnetic beads (Pierce) and incubated for 1 hr at 4° before beads were washed three times with GST buffer. Similarly, yeast cells expressing endogenous levels of VSV-tagged Rad51 (Open Biosystems) were resuspended in Rad51 buffer (50 mM Tris, pH 7.5, 0.01% NP-40, 5 mM β-glycerol phosphate, 2 mM magnesium acetate, and 120 mM NaCl) with HALT protease inhibitor cocktail (Pierce), lysed with glass beads in a BeadBeater, and cleared by centrifugation at 14,000 rpm for 20 min at 4°. Lysate was treated with benzonase (Sigma), and 10 mg of lysate were incubated with Sgs1-bound magnetic beads for 120 min at room temperature while rotating. Beads were washed five times with Rad51 buffer plus HALT protease inhibitor cocktail (Pierce) and then boiled in Laemmli buffer (Bio-Rad, Hercules, CA) for 10 min. The eluate was separated by 10% SDS-polyacrylamide gel electrophoresis. Sgs1 fragments and Rad51 were detected by western blotting using monoclonal antibodies against GST (Covance) and VSV (Sigma) epitopes.

### Data availability

Yeast strains are available upon request. Table S1 contains a list of yeast strains used in this study and detailed genotype descriptions.

## Results

### Rad51 binds to the loop that connects the helicase core of Sgs1 to the helicase- and RNaseD C-terminal (HRDC) domain

*SGS1* and *RAD51* are epistatic and the gene products interact physically (Wu *et al.* 2001; Torres *et al.* 2004). Using a yeast two-hybrid assay, the physical interaction with Rad51 was previously mapped to the last 469 residues of Sgs1 (residues 978–1447). This region flanks the ATPase domain and contains the conserved RQC domain, which is essential for the helicase activity of Sgs1, as well as other conserved sites, including the HRDC domain and an Mlh1-binding site (Pedrazzi *et al.* 2001; Wu *et al.* 2001). Thus, disrupting Rad51 binding by deleting this 469-residue region disrupts multiple other Sgs1 functions. Therefore, to enable the elucidation of the biological importance of the interaction between Sgs1 and Rad51 for HR, we sought to identify a separation-of-function mutation in Sgs1 that specifically disrupts the Rad51 interaction, but leaves other functional sites intact.

**Figure 1:**
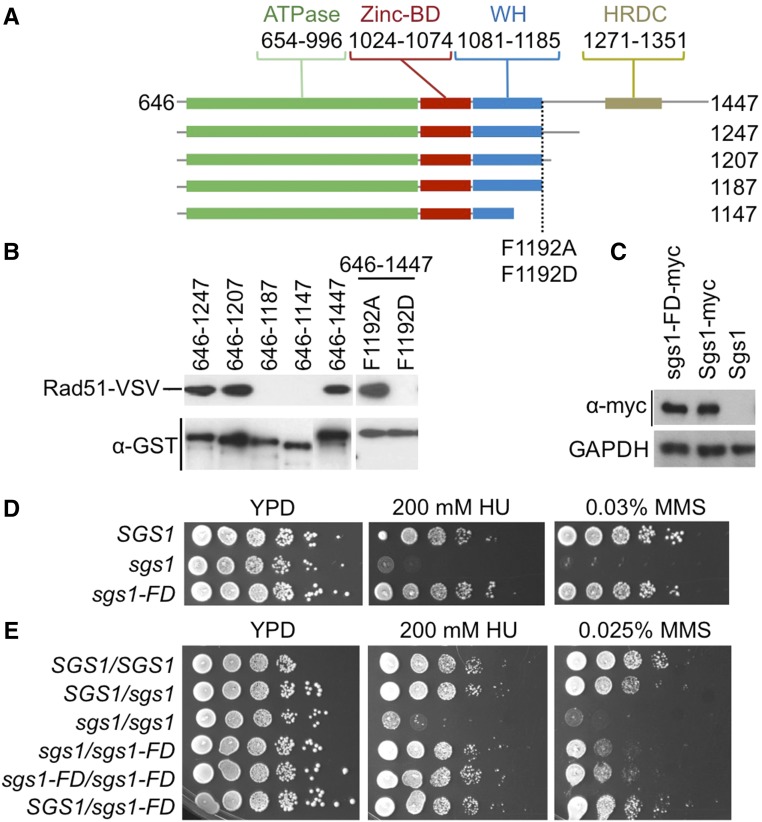
Rad51 interacts with Sgs1 downstream of the winged-helix (WH) domain. (A) Domain structure of Sgs1. The helicase core of Sgs1 consists of the ATPase domain, which is formed by two RecA-like lobes, and the RecQ-C terminal (RQC) domain, which consists of a zinc-binding (Zinc-BD) and a WH domain. The helicase- and RNaseD C-terminal (HRDC) domain is separated from the helicase core by a proline/glycine-rich loop. The 645-residue unstructured N-terminal tail is omitted. (B) Pulldown of Rad51-V5-3XVSV with GST-tagged Sgs1 fragments, and sgs1-FD and sgs1-FA mutants. GAPDH, glyceraldehyde 3-phosphate dehydrogenase. (C) Expression levels of the *sgs1-FD* allele from the chromosomal *SGS1* promoter do not differ from the wild-type *SGS1* allele. Whole-cell extracts were prepared by trichloroacetic acid extraction from an equal number of cells expressing myc epitope-tagged sgs1-FD or wild-type Sgs1, as previously described (Kennedy *et al.* 2013), and analyzed by western blot using a monoclonal (9E10) c-myc antibody (SCBT). (D) Unlike an *SGS1* deletion, the *sgs1-FD* allele does not cause hypersensitivity to MMS or HU in haploid cells. (E) Homozygosity for the *sgs1-FD* mutation causes mild sensitivity to HU and MMS in diploid cells.

To narrow down the Rad51-binding region, we purified fragments of Sgs1 as GST fusions from *E. coli* and tested their ability to pull-down endogenous VSV epitope-tagged Rad51 from yeast whole-cell extracts. We determined that deleting up to 240 C-terminal residues of Sgs1 did not impair its ability to interact with Rad51, whereas a deletion of 260 residues abolished it (Figure 1, A and B). This critical 20-residue region maps to residues 1187–1207, immediately C-terminal of the winged-helix (WH) domain, and contains a phenylalanine at position 1192. Mutating this hydrophobic residue to aspartic acid (F1192D) disrupted Rad51 binding, whereas mutating it to alanine did not have an effect (Figure 1B).

### Unlike loss of Sgs1 helicase activity, loss of Sgs1-Rad51 binding does not cause DNA damage sensitivity and genome instability in haploid cells

To determine how the loss of Rad51 binding affects Sgs1 function *in vivo*, we integrated the *sgs1-F1192D* allele (hereafter *sgs1-FD*) at the chromosomal locus under control of the *SGS1* promoter. Expression levels of sgs1-FD were similar to those of wild-type Sgs1 (Figure 1C). Unlike an *SGS1* deletion, the *sgs1-FD* mutation did not increase genome instability (Table 1) or sensitivity to HU (Figure 1D). Since the *sgs1-FD* mutation is disrupting a link between Sgs1 and HR, we also tested its effect on DNA damage sensitivity in diploids, which depend more strongly on HR for the repair of DNA breaks than haploids (Frank-Vaillant and Marcand 2001; Li and Tye 2011). Diploids were indeed more sensitive to MMS if they were homozygous for the *sgs1-FD* mutation (Figure 1D), indicating a mild DNA repair defect in the *sgs1-FD* mutant.

**Table 1:** Effect of the *sgs1-FD* mutation on the rate of accumulation of GCRs

Relevant genotype	GCR rate*^a^* (Can^r^ 5-FOA^r^ × 10^−10^)	95% C.I.*^b^* (Can^r^ 5-FOA^r^ × 10^−10^)
Wild-type	1.1	< 1–6.2
*sgs1*	71	53–104
*sgs1-FA*	< 9	< 7–9
*sgs1-FD*	< 7	< 6–8
*exo1*	14	7–28
*sgs1 exo1*	40500	31000–49900
*sgs1-FD exo1*	83	49–124
*sae2*	12	< 7–18
*sgs1-FD sae2*	306	(124–424)
*mre11*	2200	n.d.
*mre11 sgs1-FD*	2030	1170–2480
*top3*	27	17–96
*top3 sgs1-FD*	12	9–36
*rad24*	23	9–37
*sgs1 rad24*	136	117–216
*sgs1-FD rad24*	26	10–69
*pol32*	20	15–26
*sgs1 pol32*	25	< 24–105
*sgs1-FD pol32*	< 8	< 7–19
*rrm3*	14	5–28
*sgs1 rrm3*	656	311–1290
*sgs1-FD rrm3*	< 6	< 5–8
*srs2*	0.6	< 2–11
*sgs1-FD srs2*	8	< 7–11

GCR, gross chromosomal rearrangement; Can^r^, canavanine resistant; n.d., not detected.

a GCR rates for *mre11* and *top3* mutants are from Myung *et al.* (2001); for *sgs1 rrm3* from Schmidt *et al.* (2006); for *sgs1 exo1* and *exo1* from Doerfler and Schmidt (2014); and for *srs2* from Schmidt *et al.* (2010b).

b 95% C.I.s were calculated according to Nair (1940).

### Rad51 binding to Sgs1 is required for normal growth, DNA damage tolerance, and genome stability in the absence of Sae2, but not Mre11

**Figure 2:**
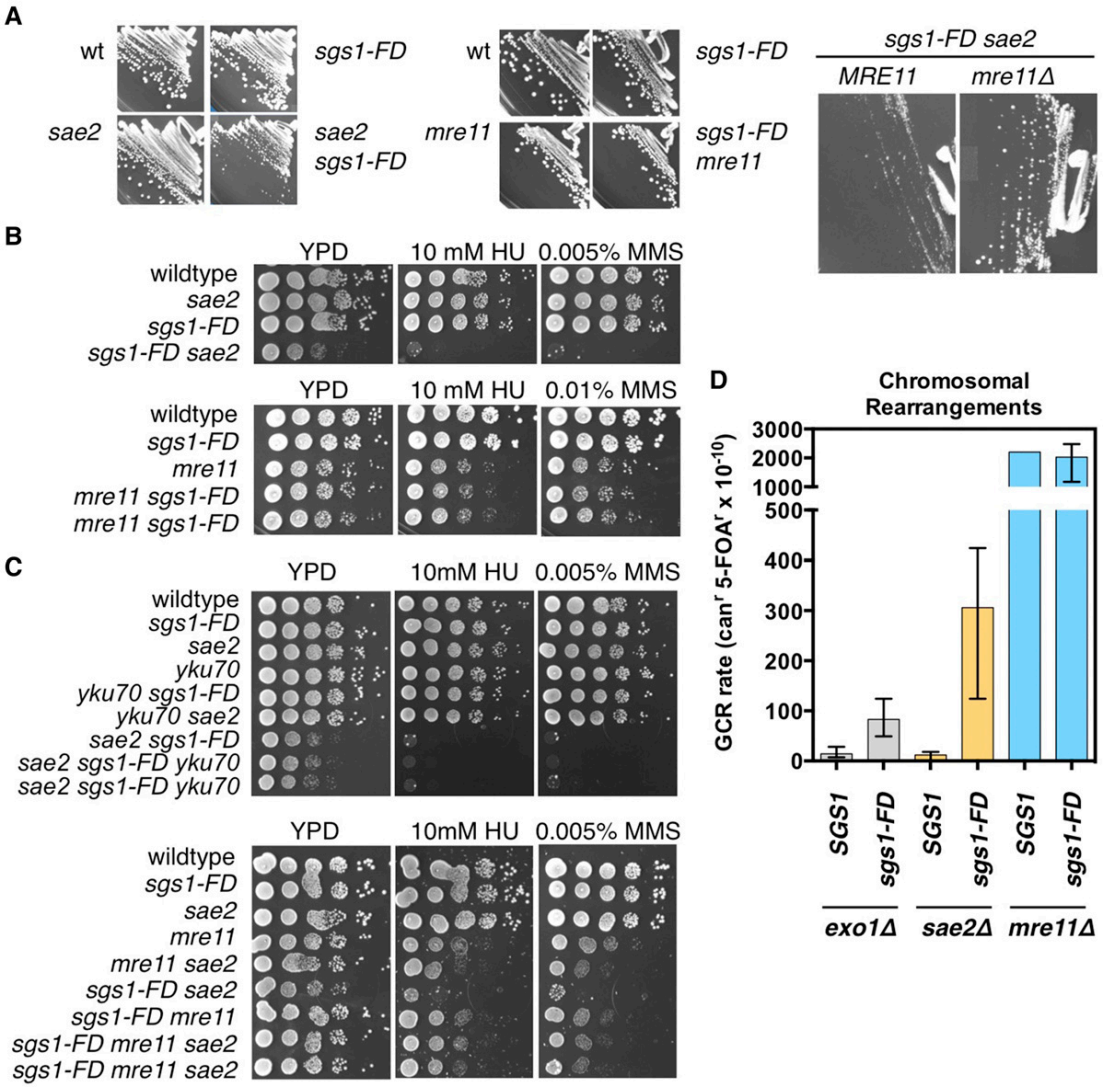
Effect of the *sgs1-FD* mutation on fitness, DNA damage sensitivity, and genome stability of *sae2*∆, *mre11*∆, and *yku70*∆ mutants. (A) *sgs1-FD* causes a severe growth defect in the *sae2*∆ mutant, but not in the *mre11*∆ mutant. Deletion of *MRE11* suppresses the growth defect of *sgs1-FD sae2∆*. wt, wild-type. (B) *sgs1-FD* increases the HU/MMS sensitivity of the *sae2*∆ mutant, but not the *mre11*∆ mutant. (C) Unlike *mre11*Δ, *yku70*Δ does not suppress the growth defect and HU/MMS hypersensitivity of the *sgs1-FD sae2∆* mutant. (D) *sgs1-FD* causes synergistic gross chromosomal rearrangement (GCR) rate increases in *sae2*∆ and *exo1*∆ mutants, but has no effect on GCR accumulation in the *mre11*∆ mutant. Median GCR rates are shown with 95% C.I.s (see also Table 1).

To identify Sgs1 functions that are impacted by its binding to Rad51, we first investigated genetic interactions between *sgs1-FD* and HR genes. In HR, Sgs1 acts in addition to Exo1 in the resection of DSBs after their initial nucleolytic processing by Sae2/MRX (Mimitou and Symington 2008). In cells lacking *SGS1*, deletion of *EXO1* causes a fitness defect and one of the largest known synergistic increases in genomic instability (> 500-fold) (Gravel *et al.* 2008; Doerfler *et al.* 2011; Doerfler and Schmidt 2014), whereas a deletion of *SAE2* or *MRE11* is synthetically lethal with *sgs1*Δ (Shor *et al.* 2002; Pan *et al.* 2006). However, these reported phenotypic similarities between Sae2 and Mre11 deficiency did not apply to the *sgs1-FD* mutant. The *sgs1-FD* mutation caused a significant fitness defect in the *sae2*Δ mutant, but had no detrimental effect on the growth of the *mre11*Δ mutant (Figure 2A). The *sgs1-FD* allele also increased hypersensitivity of the *sae2∆* mutant to HU and MMS, but exhibited a wild-type phenotype in the *mre11*Δ mutant (Figure 2, B and C). Moreover, the *sgs1-FD* allele led to a synergistic (25-fold) increase in the GCR rate in the *sae2*Δ mutant, but had no effect on the accumulation of genome rearrangements in the *mre11*Δ mutant (Figure 2D).

In the current model of DSB end processing, MRX and Sae2 bind to the unprocessed ends, trimming off a few nucleotides and causing their own release from the DNA (Mimitou and Symington 2008; Zhu *et al.* 2008). These trimmed ends are poor substrates for Ku binding, but good substrates for extensive nucleolytic processing by Exo1 and Sgs1/Dna2 to produce the long 3′ terminated overhangs for Rad51-mediated homology search (Mimitou and Symington 2008; Zhu *et al.* 2008). When initial trimming and long-range resection are impaired due to an absence of Sae2 and Sgs1, cells die (Tong *et al.* 2001; Ooi *et al.* 2003). However, these cells are rescued by deleting *YKU70*, suggesting that preventing Ku from binding to the DSB ends makes them accessible to the alternative, Exo1-mediated pathway for long-range resection, thus bypassing the requirement of Sae2 for the removal of Ku and that of Sgs1 for long-range resection (Mimitou and Symington 2010). Based on this finding, any *sgs1* mutation that causes a resection defect and a synthetic growth defect with *sae2*Δ should be suppressed by deletion of *YKU70*. Indeed, the defects of the *sgs1-D664*Δ mutant, which include a severe fitness defect with *sae2*Δ and a resection defect, are bypassed in cells lacking Ku (Bernstein *et al.* 2009, 2013). However, we observed that neither the severe fitness defect of the *sgs1-FD sae2*Δ mutant nor its DNA damage sensitivity were suppressed by deleting *YKU70* (Figure 2C), suggesting that the *sgs1-FD* mutation does not cause a resection defect.

To test the possibility that the requirement of Sae2 in the *sgs1-FD* mutant was related to an MRX-related function of Sae2, we next deleted *MRE11* in the *sae2*Δ *sgs1-FD* mutant. We observed that the *mre11*Δ mutation suppressed the growth defect and the associated HU/MMS sensitivity of the *sgs1-FD sae2*Δ mutant to levels observed in the *mre11*Δ *sae2*Δ mutant (Figure 2, A and C), indicating that, unlike in the *sgs1*Δ mutant, MRX is not required in the *sgs1-FD* mutant and that, in fact, the inability to remove MRX from the DSB ends is toxic in the *sgs1-FD* mutant.

### *sgs1-FD* increases genome instability and DNA damage hypersensitivity in cells lacking Exo1

**Figure 3:**
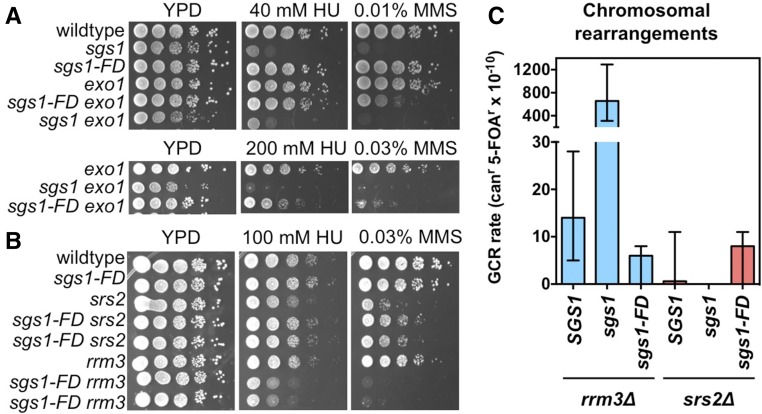
Effect of the *sgs1-FD* mutation on fitness and DNA damage sensitivity of *exo1*∆, *srs2*∆, *rrm3*∆, and *top3*∆ mutants. (A) *sgs1-FD* sensitizes the *exo1*∆ mutant to MMS and, to a lesser extent, HU. (B) *sgs1-FD* suppresses the HU/MMS sensitivity of the *srs2*∆ mutant and increases the HU/MMS sensitivity of the *rrm3*∆ mutant. (C) Unlike deletion of *SGS1*, *sgs1-FD* does not cause a gross chromosomal rearrangement (GCR) rate increase in the *rrm3*∆ mutant. *sgs1-FD* does not affect GCR formation in the *srs2∆* mutant (see also Table 1). Median GCR rates are shown with 95% C.I.s.

The need in the *sgs1-FD* mutant for *SAE2* prompted us to further investigate the requirement of *EXO1*, which cooperates with Sgs1/Dna2 during more extensive resection of DSB after Sae2. The *exo1*Δ mutant is mildly sensitive to high concentrations of MMS, but not HU (Doerfler and Schmidt 2014). The *sgs1-FD* allele increased the sensitivity of the *exo1*Δ mutant to MMS and caused sensitivity to 200 mM HU, but remained far below the effect of an *SGS1* deletion (Figure 3A). The *sgs1-FD* allele also caused a significant (sixfold) increase in the accumulation of genome rearrangements in the *exo1*Δ mutant (Figure 2D), albeit this was also much milder than the 500-fold increase in GCR accumulation previously reported for the *SGS1* deletion (Gravel *et al.* 2008; Doerfler *et al.* 2011).

### Suppression of the severe growth defect of the *top3*Δ mutant by the *sgs1-FD* mutation

In addition to interacting with Dna2 and RPA during DSB resection, Sgs1 forms a complex with Top3/Rmi1 to dissolve dHJs (Gangloff *et al.* 1994; Chang *et al.* 2005; Mullen *et al.* 2005). *In vitro*, Top3 also stimulates Sgs1 activity in DSB resection and resolves protein-bound D-loops (Cejka *et al.* 2010a; Fasching *et al.* 2015). Both deletion of *SGS1* or loss of Sgs1 helicase activity suppress the severe growth defect of the *top3*Δ mutant, which has been interpreted to mean that Sgs1 produces HR intermediates that then require Top3 for dissolution (Gangloff *et al.* 1994). We found that the *sgs1-FD* allele also suppressed the severe slow-growth phenotype of the *top3*Δ mutant to a similar extent as the *sgs1*Δ or *sgs1-HD* alleles, and improved growth during exposure to HU or MMS (Figure 3, A and B). This suggests that the interaction between Sgs1 and Rad51 drives the formation of recombination intermediates and thereby significantly contributes to the severe growth defect of the *top3*Δ mutant.

**Figure 4:**
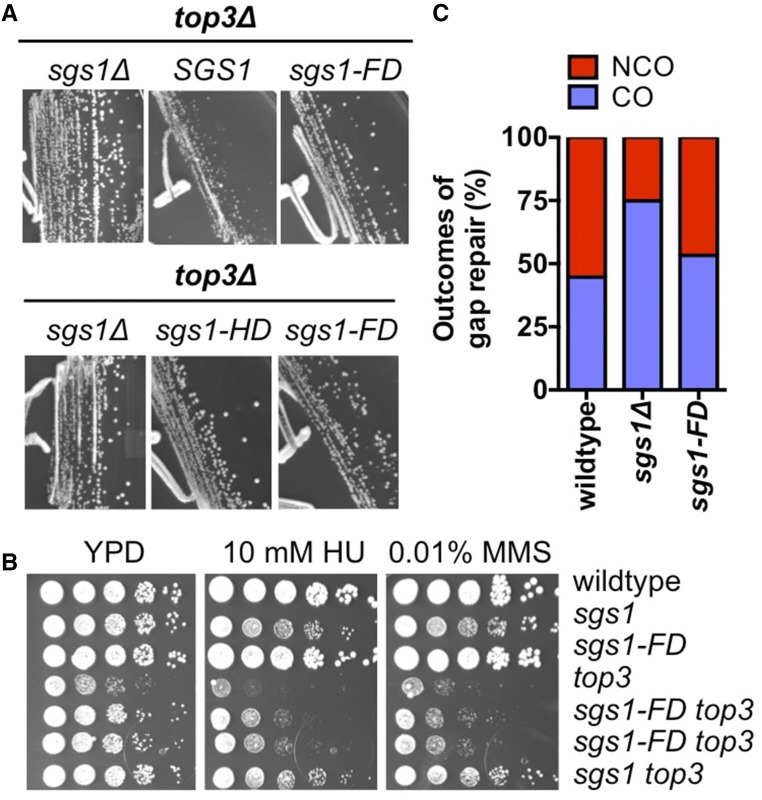
Disrupting Sgs1-Rad51 interaction suppresses the severe growth defect of the top3 mutant, but has no effect on crossover (CO)/noncrossover (NCO) formation. (A) *sgs1-FD* suppresses *top3*∆ slow growth nearly as effectively as a deletion of *SGS1* or helicase-dead *sgs1-HD*. (B) Improved growth of *sgs1-FD top3∆* in the presence of HU and MMS correlates with partial suppression of the *top3*∆ fitness defect by *sgs1-FD*. (C) In contrast to an *SGS1* deletion, the *sgs1-FD* mutation does not have a major effect on the ratio between CO/NCO outcomes in gap repair. Over 140 His^+^ transformants, each from two independent *can1*::*his3-0*,*∆3′* isolates of the wild-type, *sgs1-FD*, and *sgs1∆* strains were analyzed.

Since Sgs1 and its interaction with Top3/Rmi1 are important for the dissolution of recombination intermediates, we also tested the effect of the *sgs1-FD* mutation on crossover and noncrossover formation in a gap repair assay (Welz-Voegele and Jinks-Robertson 2008). Deletion of *SGS1* led to an increase in the fraction of crossovers, which is consistent with previous findings (Welz-Voegele and Jinks-Robertson 2008), whereas the *sgs1-FD* mutant exhibited a ratio of crossovers to noncrossovers similar to that of wild-type cells (Figure 4C). This suggests that the ability of Sgs1/Top3/Rmi1 to dissolve HJs is largely unaffected by the FD mutation in Sgs1.

### Opposite effects of the *sgs1-FD* mutation on the DNA damage sensitivity of *srs2*Δ and *rrm3*Δ mutants

Srs2 acts as an inhibitor of HR through its ability to disrupt the Rad51 presynaptic filament (Krejci *et al.* 2003; Veaute *et al.* 2003). In the absence of Srs2, cells become hyperrecombinogenic, hypersensitive to exogenous DNA damage and replication stress, and dependent on Sgs1 for viability (Lee *et al.* 1999; Krejci *et al.* 2003; Veaute *et al.* 2003). The negative genetic interactions of the *sgs1-FD* allele with *sae2*Δ and *exo1*Δ mutations, and the positive interaction with *top3*Δ, suggest that *sgs1-FD* is a hyporecombination allele of *SGS1*. To further explore this possibility, we introduced *sgs1-FD* into the *srs2*Δ mutant, which we expected to benefit from a reduction in HR. Indeed, in stark contrast to a deletion of *SGS1* or loss of Sgs1 helicase activity, which are both lethal to *srs2*Δ cells, the *sgs1-FD* allele had no detrimental effect on the growth of *srs2*Δ cells and, in fact, suppressed the hypersensitivity of *srs2*Δ cells to HU and MMS by > 10-fold (Figure 3B), consistent with *sgs1-FD* being a hyporecombination allele.

We also investigated the importance of the Sgs1-Rad51 interaction in the *rrm3*Δ mutant. Replisomes pause frequently at many sites throughout the genome when the Rrm3 helicase is absent, generating DNA lesions that are substrates for Sgs1- and Rad51-dependent repair (Ivessa *et al.* 2002; Schmidt and Kolodner 2004; Torres *et al.* 2004). Like a deletion of *SRS2*, deletion of *RRM3* causes a severe growth defect in *sgs1*Δ cells that can be suppressed by deleting *RAD51* (Schmidt and Kolodner 2004; Torres *et al.* 2004). The *sgs1-FD* mutation did not cause a growth defect in *rrm3*Δ cells; however, cells became highly sensitive to both HU and MMS (Figure 3B). Despite the increased DNA damage sensitivity, the *rrm3*Δ *sgs1-FD* mutant did not accumulate genome rearrangements (Figure 3C and Table 1), in contrast to the *rrm3*Δ *sgs1*Δ mutant (Schmidt *et al.* 2006). This genetic interaction between *rrm3*Δ and a hypomorphic allele of *SGS1* further underscores the strong dependence of the repair of the replication-associated DNA lesions in *rrm3*Δ cells on homology-directed replication fork restart and rescue (Syed *et al.* 2016).

### Sgs1-Rad51 interaction promotes large deletions and contributes to DNA damage hypersensitivity of cells lacking Pol32

**Figure 5:**
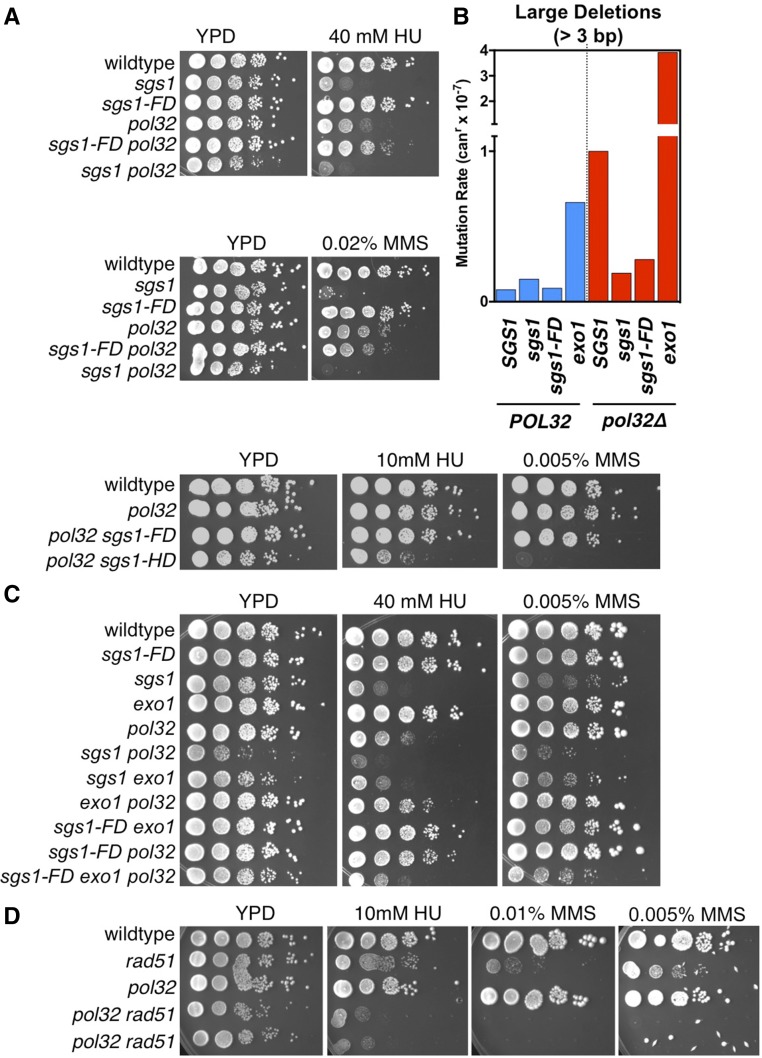
Effect of *sgs1-FD* on DNA damage sensitivity and large deletion formation in the absence of Pol32. (A) Suppression of HU sensitivity of the *pol32∆* mutant by *sgs1-FD*. In contrast, *SGS1* deletion or helicase-defective *sgs1-HD* increase DNA damage sensitivity of *pol32*Δ cells. (B) Accumulation of large deletions (> 3 bp) in *CAN1*, characteristic of cells lacking the Pol32 subunit of polymerase δ, is suppressed by *sgs1-FD* or by deletion of *SGS1*, but stimulated by deletion of *EXO1*. (C) Whereas *exo1*∆ and *sgs1-FD* suppress HU sensitivity of the *pol32*∆ mutant, combination of both *exo1∆* and *sgs1-FD* mutations increases DNA damage sensitivity of the *pol32*∆ mutant. (D) Deletion of *RAD51* in the *pol32∆* mutant causes severe hypersensitivity to HU and MMS.

In the absence of *POL32*, which connects polymerase δ to the processivity factor PCNA, DNA replication is inefficient and prone to pausing and mutations (Burgers and Gerik 1998; Huang *et al.* 2002; Johansson *et al.* 2004). Since an *SGS1* deletion causes a fitness defect and increased HU and MMS sensitivity in the *pol32*Δ mutant, we decided to assess the effect of the *sgs1-FD* allele in this mutant. Surprisingly, we found that the *sgs1-FD* mutation had the opposite effect of the *SGS1* deletion and the helicase-defective *sgs1-HD* allele, suppressing the HU hypersensitivity of the *pol32*Δ mutant (Figure 5A). Since the HU hypersensitivity of the *pol32*Δ mutant is also suppressed by deletion of *EXO1* (Doerfler and Schmidt 2014), we next tested the combined effect of *exo1*Δ and *sgs1-FD* mutations on HU sensitivity of the *pol32*Δ mutant (Figure 5B). However, instead of suppression, sensitivity to HU and MMS increased even at low drug concentrations, suggesting that Exo1 and the Sgs1-Rad51 interaction cooperate in a pathway that is required in the absence of Pol32. Since both Sgs1 and Exo1 act in DSB end processing to initiate HR, we investigated the effect of a *RAD51* deletion on the HU/MMS sensitivity of the *pol32*Δ mutant and observed a strong increase in sensitivity (Figure 5B). This suggests that *pol32*Δ cells depend on a Rad51-dependent HR pathway for the survival of replication stress, and that Exo1 and the Sgs1-Rad51 interaction independently promote this pathway.

The accumulation of large deletions between short repeats in *CAN1* or other genomic loci is characteristic of *pol32*Δ cells, and has been explained by an increased propensity of the nascent strands to dissociate from their templates as a result of frequent pausing, followed by error-prone reannealing (Huang *et al.* 2002). When we combined the *pol32*Δ mutation with the *sgs1-FD* mutation there was no significant change in the mutation rate at *CAN1* (Table S2), but the rate of large deletions was reduced fourfold. This reduction is similar to the sixfold reduction when *SGS1* is deleted, suggesting that the interaction of Sgs1 with Rad51 contributes to the formation of large deletions in the absence of Pol32 (Figure 5C). Deletion of *EXO1* in the *pol32*Δ mutant had the opposite effect, increasing the rate of large deletions fourfold (Figure 5C). These observations indicate that, in contrast to their cooperative roles in DSB end resection, Exo1 and Sgs1 have opposite effects at impaired replication forks. For example, Exo1 might prevent deletions by degrading the nascent DNA strands that are prone to DNA slippage, whereas Sgs1 and the Sgs1-Rad51 interaction might help generate deletions by facilitating slipped-strand mispairing at downstream repeats.

### Rad52/Rad59-mediated DNA repair, but not Rad51, is essential for cells with compromised DNA resection due to a lack of Sgs1 and Exo1

**Figure 6:**
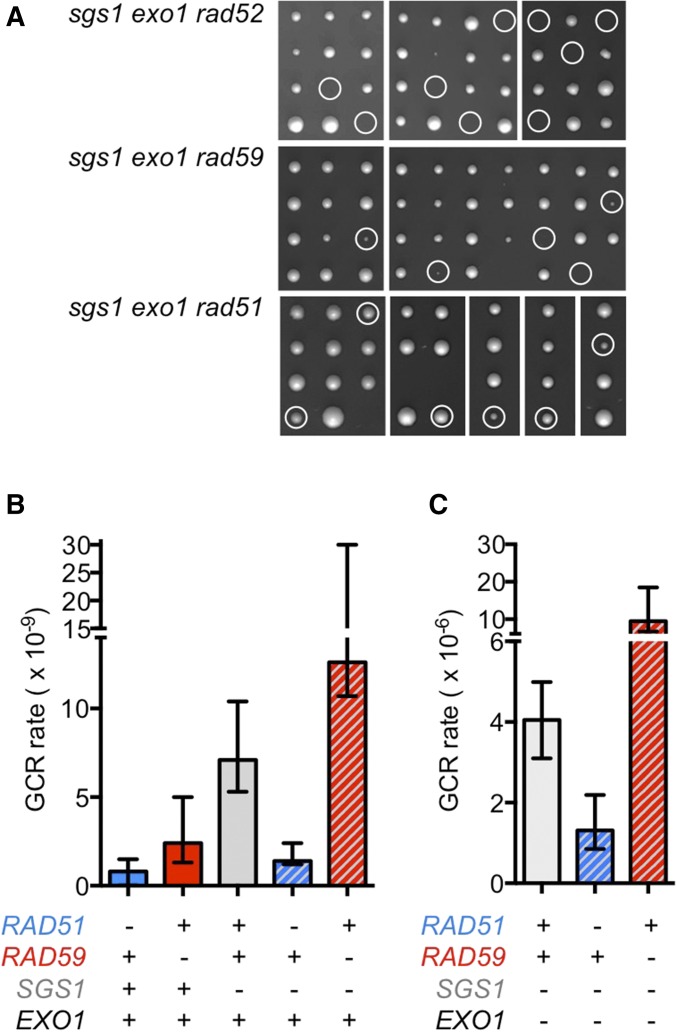
Effect of *RAD52*, *RAD51*, and *RAD59* deletions on viability and genome stability of the *sgs1∆ exo1∆* mutant. (A) As shown by tetrad dissections, deletion of *RAD52* in the *sgs1∆ exo1∆* mutant is lethal, and deletion of *RAD59* causes a severe growth defect. In contrast, deletion of *RAD51* causes only a mild growth defect. Triple-mutant spores are indicated by a white circle. (B) Deletion of *RAD51* suppresses gross chromosomal rearrangements (GCRs) in the *sgs1*∆ mutant, whereas deletion of *RAD59* stimulates GCR formation in the *sgs1*∆ mutant. (C) Deletion of *EXO1* increases GCR formation in the *sgs1*∆ mutant ∼700-fold, but does not affect the genetic interactions of *sgs1∆* with *rad51∆* and *rad59*∆. Median GCR rates are shown with 95% C.I.s.

Cells lacking Sgs1 and Exo1 show minimal resection of DSBs and accumulate GCRs at an extreme level (Gravel *et al.* 2008; Mimitou and Symington 2008; Doerfler *et al.* 2011). Because of the failure to sufficiently resect DSB ends, we expected that DSBs would not be suitable for repair by HR. However, surprisingly, we found that deleting *RAD52* was lethal in the *sgs1*Δ *exo1*Δ mutant, and deletion of *RAD59* caused an extreme growth defect (Figure 6A). In contrast, deleting *RAD51* caused only a mild fitness defect (Figure 6A), consistent with the ability of other groups to readily obtain and characterize the *sgs1*Δ *exo1*Δ *rad51*Δ mutant (Mimitou and Symington 2008; Bernstein *et al.* 2013; Signon and Simon 2014).

When we analyzed the effect of HR mutations on chromosome rearrangements in the *sgs1*Δ *exo1*Δ mutant, we found that deleting *RAD51* significantly suppressed (3.1-fold) their accumulation (Figure 6C and Table 2). We also analyzed GCR formation in the *sgs1*Δ *exo1*Δ *rad59*Δ mutant. Because of the severe growth defect of this mutant and the associated risk of obtaining suppressors during prolonged propagation, we set up all cell cultures from colonies immediately after they formed from meiotic products of the heterozygous diploid. In contrast to the decrease upon *RAD51* deletion, *RAD59* deletion doubled (2.4-fold) the GCR rate of *sgs1*Δ *exo1*Δ cells (Figure 6C and Table 2).

**Table 2:** Differential effects of *RAD51* and *RAD59* deletions on GCR formation in *sgs1* and *sgs1 exo1* mutants

Genotype	GCR rate*^a^* (Can^r^ 5-FOA^r^ × 10^−10^)	95% C.I.*^b^* (Can^r^ 5-FOA^r^ × 10^−10^)
Wild-type	1.1	< 1–6.2
*sgs1*	71	53–104
*exo1*	14	7–28
*rad51*	< 8	< 7–15
*rad59*	24	13–50
*rad52*	138	16–267
*sgs1 exo1*	40500	31000–49900
*sgs1 rad51*	14	12–24
*sgs1 rad59*	126	107–300
*sgs1 rad52*	308	140–452
*sgs1 rad51 exo1*	13100	8520–21900
*sgs1 rad59 exo1*	94900	67400–185000

GCR, gross chromosomal rearrangement; Can^r^, canavanine resistant.

a GCR rate for *sgs1 rad59* is from Doerfler *et al.* (2011).

b 95% C.I.s were calculated according to Nair (1940).

The dramatic decrease in viability of the *sgs1*Δ *exo1*Δ mutant upon *RAD59* and *RAD52* deletion suggests that a Rad59-dependent HR pathway repairs DNA lesions in this mutant. The decrease in GCR formation upon *RAD51* deletion and the opposite effect of a *RAD59* deletion further suggest that Rad51 and Rad59 compete for repair of these incompletely processed DNA lesions in the *sgs1*Δ *exo1*Δ mutant, and that repair by Rad51, but not Rad59, is mutagenic.

Interestingly, we observed that the effect of *RAD51* and *RAD59* deletions on the accumulation of GCRs is the same in *sgs1*Δ cells with *EXO1* intact, as in cells with *EXO1* deleted; that is, a *RAD51* deletion led to a significant (5.1-fold) decrease in the GCR rate of *sgs1*Δ cells (*vs.* 3.1-fold decrease in *sgs1*Δ *exo1*Δ) and *RAD59* deletion to a significant (1.8-fold) increase (*vs.* a 2.3-fold increase in *sgs1*Δ *exo1*Δ) (Figure 6, B and C and Table 2). Essentially, deleting *EXO1* increased genome instability ∼700-fold, but had no effect on the genetic interactions between *sgs1*Δ, *rad51*Δ, and *rad59*Δ (compare the last three columns in Figure 6B with Figure 6C).

## Discussion

We have identified a novel separation-of-function mutant of Sgs1 (sgs1-FD) that fails to interact with Rad51, but does not cause the severe sensitivity to DNA-damaging agents seen in cells lacking Sgs1 or expressing helicase-defective Sgs1. Novel positive and negative genetic interactions between this *sgs1-FD* allele and mutations in genes with roles in HR (*mre11*Δ, *sae2*Δ, *srs2*, *exo1*Δ, and *top3*Δ) or replisome progression (*pol32*Δ and *rrm3*Δ) suggest that the physical interaction between Sgs1 and Rad51 stimulates homology-dependent DNA repair.

We observed the strongest genetic interaction of the *sgs1-FD* allele with a *SAE2* deletion (Figure 2). Sae2 removes MRX from DSB ends and prevents Ku binding, making the DSB accessible to extensive resection by Sgs1/Dna2 and Exo1 (Mimitou and Symington 2010). *YKU70* deletion suppresses resection defects in cells that lack Sae2 and Sgs1 activities by allowing the alternative Exo1 pathway access to the DSB ends for resection (Bernstein *et al.* 2009, 2013; Mimitou and Symington 2010; Shim *et al.* 2010). In addition to suppressing the DNA damage sensitivity and fitness defect of the *sgs1*Δ *sae2*Δ mutant, the resection-defective *sgs1-D664*Δ mutant was found to benefit from deleting *YKU70* (Bernstein *et al.* 2009, 2013). In contrast, deleting *YKU70* had no effect on the *sgs1-FD sae2*Δ mutant identified here, indicating that the *sgs1-FD* mutant does not benefit from increased Exo1 activity at DSBs and, thus, that *sgs1-FD* is proficient for resection.

Surprisingly, even though Sae2 acts with MRX in the initial processing step, and *sgs1*Δ is synthetically lethal with both *sae2*Δ and *mre11*Δ, disruption of the Sgs1-Rad51 interaction was not detrimental to *mre11*Δ cells. In fact, the *MRE11* deletion suppressed the detrimental effects of the *sgs1-FD* mutation in *sae2*Δ cells, suggesting that the Sae2 function that is critical in *sgs1-FD* cells is the removal of MRX from DSB ends. If MRX stays bound, the DNA damage checkpoint is activated and oligomers of the Rad9 checkpoint adaptor accumulate nearby (Usui *et al.* 2001; Clerici *et al.* 2006; Chen *et al.* 2015; Puddu *et al.* 2015). Disrupting the DNA damage checkpoint alleviates the requirement for Sae2 at DSBs (Ferrari *et al.* 2015). Although this suggests that Sgs1 can compensate for the lack of initial resection by Sae2, more extensive resection and Rad51 filament formation are still impaired by MRX stuck on the DSB ends (Ferrari *et al.* 2015; Gobbini *et al.* 2015). Recent findings have indicated that Sgs1 can eventually remove MRX and analysis of the *sgs1-D664*Δ mutant linked this ability to long-range resection by Sgs1 (Bernstein *et al.* 2013; Ferrari *et al.* 2015). Because of the resection defect of the *sgs1-D664*Δ mutant, defects of the *sgs1-D664*Δ *sae2*Δ mutant could be suppressed by deleting *YKU70*. That the *YKU70* deletion had no effect on the *sgs1-FD sae2*Δ mutant indicates that the suppression by *MRE11* deletion is not related to a resection defect in the *sgs1-FD* mutant. Therefore, we propose that the disruption of Sgs1-Rad51 interaction by the *sgs1-FD* mutation reduces the efficiency of Rad51 filament formation, and thus repair by HR. Removing *MRE11* from the DSB ends and, consequently, preventing Rad9 accumulation around the DSB ends could compensate for this deficiency in the *sae2*Δ *sgs1-FD* mutant, by increasing the efficiency of long-range resection due to increased access of sgs1-FD/Dna2 to the DSB ends. Thus, when DNA end processing is impaired because of the lack of Sae2, and persistent MRX binding and resulting checkpoint activation inhibit Sgs1/Dna2 function in resection, HR increasingly depends on the stimulation of Rad51 filament formation by Sgs1.

The requirement of Sae2 in the *sgs1-FD* mutant could also point to some overlap between the functions of Sgs1 and Sae2 during the early steps of HR, such as initial resection of DSB ends.

All other genetic interactions of the *sgs1-FD* allele investigated here are also in agreement with a role of the Sgs1-Rad51 interaction in stimulating HR, such as the positive interactions of the *sgs1-FD* mutation with *top3*Δ, *pol32*Δ, and *srs2*Δ, and the negative interaction with *rrm3*Δ. The suppression of the DNA damage sensitivity of the *srs2*Δ mutant particularly strengthens our hypothesis that the Sgs1-Rad51 interaction stimulates Rad51 filament formation. Based on the ability of Srs2 to disassemble Rad51 filaments (Krejci *et al.* 2003), suppression of *srs2*Δ *sgs1*Δ synthetic lethality by *RAD51* deletion (Gangloff *et al.* 2000) could be interpreted in two ways: either Sgs1 acts like Srs2 by disassembling presynaptic Rad51 filaments, or Sgs1 in complex with Top3/Rmi1 is needed to dissolve the accumulating recombination intermediates that overwhelm the cell because Rad51 filaments are no longer disrupted by Srs2. Our findings suggest the second explanation to be true; if the Sgs1-Rad51 interaction indeed promoted the disassembly of presynaptic Rad51 filaments, then the disruption of the Sgs1-Rad51 interaction by the *sgs1-FD* mutation would not have suppressed the DNA damage sensitivity of the *srs2*Δ mutant (Figure 3B).

**Figure 7:**
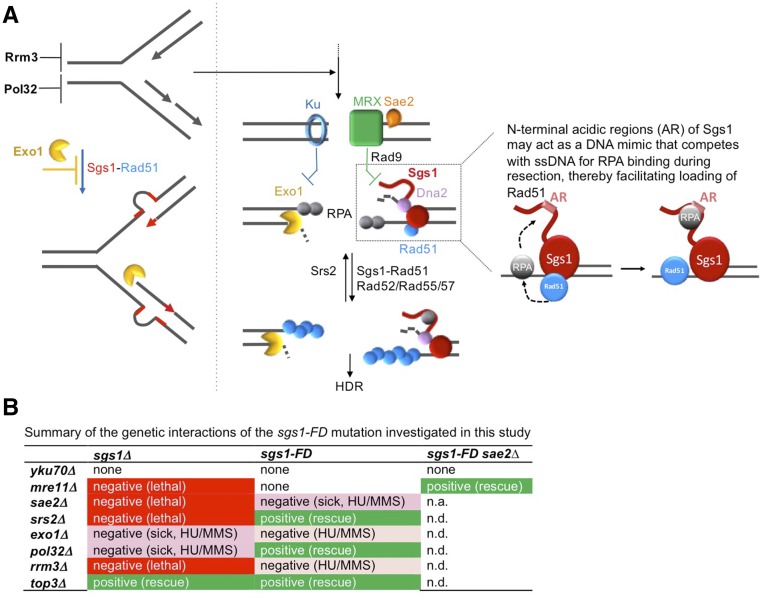
Model for a stimulatory role of the Sgs1-Rad51 interaction in homology-dependent repair (HDR) of spontaneous DNA lesions. (A) Replication stress, exogenous DNA damage, or disruption of factors with roles in replisome progression (*e.g.*, *rrm3∆* and *pol32∆*) can impair replication forks and give rise to mutations (left) and double-strand breaks (DSBs) (right). Right panel: unprocessed DNA breaks can be bound by Ku and MRX. Nuclease activity of MRX/Sae2 trims the ends, which are then extensively resected by Exo1 and Sgs1/Dna2. Bound Ku and MRX inhibit long-range resection by Exo1 and Sgs1/Dna2, respectively. As the *sgs1-FD* mutant does not benefit from *YKU70* deletion, suggesting it does not have a significant resection defect, we propose that the Sgs1-Rad51 interaction could instead stimulate homologous recombination (HR) by linking Sgs1’s role in long-range resection to Rad51 filament formation. Specifically, the acidic regions (AR) in the unstructured N-terminal tail of Sgs1, through their capacity to bind RPA, could act as a DNA mimic, allowing Sgs1 to compete with single-stranded DNA (ssDNA) for RPA binding, thereby facilitating deposition of Sgs1-bound Rad51 onto ssDNA during resection. Note: Ku, MRX, Exo1, and Sgs1/Dna2 can act on the same DSB end; two ends are shown to separate their activities for clarity only. Left panel: in the absence of Pol32, cells are known to accumulate large deletions of sequences flanked by direct short repeats. We propose a model whereby Sgs1, through its interaction with Rad51, stimulates the formation of these deletions (Figure 5B) by mediating misannealing of the nascent strands with downstream repeated sequences, whereas the 5′–3′ exonuclease Exo1 reduces deletion formation (Figure 5B) by degrading nascent DNA on the lagging strand from its accessible 5′ end. (B) Summary of differential genetic interactions of the *sgs1-FD* allele and the *SGS1* deletion with mutations in DNA recombination and replication factors. n.a., not applicable; n.d., not determined.

Thus, taken together, the genetic interactions of the *sgs1-FD* allele are distinct from those of the *sgs1*∆ and helicase-defective *sgs1-hd* alleles (Figure 7B), and are consistent with a model (Figure 7A) whereby Sgs1 is not only responsible for the resection of DSB ends and the formation of ssDNA overhangs but, through interaction with Rad51, promotes HR by stimulating formation of the Rad51 presynaptic filament. As DNA ends are resected, RPA binding to the newly formed ssDNA overhangs limits the initiation of the Rad51 filament. Rad52 is essential to overcome this limitation and form a productive Rad51 presynaptic filament on RPA-coated ssDNA. Sgs1 binds RPA via an acidic region just upstream of the helicase core (Hegnauer *et al.* 2012). However, the biological significance of this interaction has remained unclear. We propose that the acidic region in the N-terminus of Sgs1 to which RPA binds serves as a DNA mimic, and that via this DNA mimic, Sgs1 can compete with the ssDNA overhang for RPA binding, thereby freeing up ssDNA locally for Rad51 and stimulating filament initiation (Figure 7A). This model is supported by the overlap between the distinct phenotype of the *sgs1-FD* mutant and phenotypes of mutations in the acidic region of Sgs1, including suppression of *top3∆* slow growth, wild-type-level resistance to HU and MMS, lack of a hyperrecombination phenotype, and lack of synthetic lethal interactions characteristic of the *sgs1∆* mutation (Bernstein *et al.* 2009, 2013). Such a role for Sgs1 is also reminiscent of the function of *E. coli* RecBCD, not only in resection, but also in assembling the RecA filament (Anderson and Kowalczykowski 1997). There is also *in vitro* evidence that the BLM-hRad51 interaction may play a role in loading hRad51 onto ends resected by BLM and Exo1 (Nimonkar *et al.* 2008). Moreover, the ability of BRCA2 to load Rad51 onto ssDNA *in vitro* was recently shown to be aided by interaction with a protein, DSS1, that appears to act as a DNA mimic and targets RPA on ssDNA (Zhao *et al.* 2015).

We also observed a stimulatory effect of the Sgs1-Rad51 interaction on the formation of the direct repeat-mediated large deletions characteristic of *pol32*Δ cells. However, unlike in DSB resection, Sgs1 had the opposite effect of Exo1: the Sgs1-Rad51 interaction promoted the deletions, and Exo1 suppressed them, both to approximately the same extent. The large deletions in *pol32*Δ cells most likely form during inefficient replisome progression, which makes the nascent DNA strands prone to dissociation, followed by misannealing at a repeated downstream sequence, thus deleting the sequence between the repeats. We propose a model whereby Exo1 prevents large deletions through its ability to degrade the nascent lagging DNA strand at stalled forks (Engels *et al.* 2011), and the Sgs1-Rad51 interaction, in contrast, promotes annealing of dissociated nascent strands with downstream repeated sequences (Figure 7A).

Finally, our study also provides new insight into the repair of DSBs in cells where long-range resection by Sgs1/Dna2 and Exo1 is disrupted. It is thought that for HR, the ends need to be resected extensively by Sgs1/Dna2 or Exo1 before a productive Rad51 filament can form and initiate a homology search. Hence, *sgs1∆ exo1∆* mutants should not be able to rely on HR as a major pathway for DNA lesion repair. It was therefore surprising that *sgs1∆ exo1∆* cells depend on *RAD52* for their survival. That the fitness of these cells was more dependent on Rad59 than Rad51 suggests that the minimally resected DSB ends in *sgs1*Δ *exo1*Δ cells are mainly repaired by Rad59/Rad52-dependent HR. This is consistent with a preference of Rad59 for the repair of short substrates, including by Rad51-independent break-induced replication (Sugawara *et al.* 2000; Ira and Haber 2002; Pannunzio *et al.* 2008). Interestingly, we also found that Rad59 suppressed genome rearrangements in *sgs1∆ exo1∆* mutants, whereas Rad51 increased them, suggesting that both Rad51 and Rad59 can act on minimally resected ends, but with Rad59 leading to proper repair, whereas Rad51 is mutagenic. That the genetic interactions between *SGS1*, *RAD51*, and *RAD59* were the same in the presence or absence of *EXO1*—that is, Rad59 suppressed GCRs in the absence of Sgs1 whereas Rad51 generated them—further indicates that *sgs1*Δ and *sgs1*Δ *exo1*Δ cells simply differ in the abundance of the lesions, but that the lesions are of the same type and accessed in the same manner by Rad59 and Rad51 whether Exo1 is present or not.

In addition to Rad51, Sgs1 interacts with numerous other DNA repair factors, including Top2, Top3, RPA, Mre11, Rad16, and Mlh1, and the checkpoint kinase Rad53. However, determining the significance of these interactions for Sgs1 function has remained challenging due to the lack of point mutations that disrupt individual interactions. Identifying the binding sites on Sgs1 for these other interacting partners will allow us to further dissect the well-characterized, but pleiotropic, effect of an *SGS1* deletion on DNA break repair and provide a more precise understanding of the specific roles of Sgs1 in promoting genome integrity.

## 

## Supplementary Material

Supplemental material is available online at www.genetics.org/lookup/suppl/doi:10.1534/genetics.117.300545/-/DC1.
