# Supplementary material for: Sgs1 Binding to Rad51 Stimulates Homology-Directed DNA Repair in Saccharomyces cerevisiae
Source: Genetics. 2017 Nov 21;208(1):125–38. doi: 10.1534/genetics.117.300545 (PMC5753853; doi:10.1534/genetics.117.300545)
Supplement: Supplementary file 1 [file 125TableS1.pdf]

TABLE S1. *Saccharomyces cerevisiae* strains used in this study

Strain ID[a]	Genotype
KSHY802	*MATa, ura3-52, leu2Δ1, trp1Δ63, his3Δ200, lys2Bgl, hom3-10, ade2Δ1, ade8, hxt13::URA3*
KHSY978	*MATa, ura3-52, trp1∆63, his3∆200, leu2∆1, lys2Bgl, hom3-10, ade2∆1, ade8, hxt13::URA3, srs2::G418*
KHSY1256	*MATa, ura3-52, trp1∆63, his3∆200, leu2∆1, lys2Bgl, hom3-10, ade2∆1, ade8, hxt13::URA3, rrm3::G418*
KHSY1338	*MATa, ura3-52, trp1∆63, his3∆200, leu2∆1, lys2Bgl, hom3-10, ade2∆1, ade8, hxt13::URA3, sgs1::TRP1*
KHSY2295	*MATa, ura3-52, trp1∆63, his3∆200, leu2∆1, lys2Bgl, hom3-10, ade2∆1, ade8, hxt13::URA3, rad59::G418, sgs1::TRP1*
KHSY2333	*MATa, ura3-52, trp1∆63, his3∆200, leu2∆1, lys2Bgl, hom3-10, ade2∆1, ade8, hxt13::URA3, pol32::loxP-G418-loxP*
KHSY2338	*MATa, ura3-52, trp1∆63, his3∆200, leu2∆1, lys2Bgl, hom3-10, ade2∆1, ade8, hxt13::URA3, exo1::loxP-G418-loxP*
KHSY2385	*MATα, ura3∆0, leu2∆0, his3∆1, lys2∆0, RAD51.V5.3xVSV.KANMX6*
KHSY2402	*MATa, ura3-52, trp1∆63, his3∆200, leu2∆1, lys2Bgl, hom3-10, ade2∆1, ade8, hxt13::URA3, exo1::loxP-G418-loxP, sgs1::TRP1*
KHSY2437	*MATa, ura3-52, trp1∆63, his3∆200, leu2∆1, lys2Bgl, hom3-10, ade2∆1, ade8, hxt13::URA3, pol32::loxP-G418-loxP, sgs1::TRP1*
KHSY4458	*MATa, ura3-52, trp1∆63, his3∆200, leu2∆1, lys2Bgl, hom3-10, ade2∆1, ade8, hxt13::URA3, exo1::loxP-G418-loxP, pol32::loxP-G418-loxP*
KHSY4484	*MATa, ura3-52, trp1∆63, his3∆200, leu2∆1, lys2Bgl, hom3-10, ade2∆1, ade8, hxt13::URA3 exo1::loxP-G418-loxP, rad59::G418*
KHSY4716	*MATa, ura3-52, trp1∆63, his3∆200, leu2∆1, lys2Bgl, hom3-10, ade2∆1, ade8, hxt13::URA3, top3::G418*
KHSY4800	*ura3-52/ura3-52, trp1Δ63/trp1Δ63, hisΔ200/hisΔ200, EXO1/exo1::HIS3, RAD52/rad52::URA*
KHSY4805	*MATα, ura3-52, trp1Δ63, hisΔ200, exo1::HIS3, rad52::URA*
KHSY4810	*ura3-52/ura3-52, trp1Δ63/trp1Δ63, hisΔ200/hisΔ200, EXO1/exo1::HIS3, RAD52/rad52::URA, SGS1/sgs1::TRP1*
KHSY5049	*MATa, ura3-52, trp1Δ63, hisΔ200, rad59::HIS3*
KHSY5051	*MATα, ura3-52, trp1Δ63, hisΔ200, exo1::HIS3, rad59::HIS3*
KHSY5052	*ura3-52/ura3-52, trp1Δ63/trp1Δ63, hisΔ200/hisΔ200, EXO1/exo1::HIS3, RAD59/rad59::HIS3*
KHSY5055	*MATa, ura3-52, trp1Δ63, hisΔ200, rad51::HIS3*
KHSY5056	*ura3-52/ura3-52, trp1Δ63/trp1Δ63, hisΔ200/hisΔ200, EXO1/exo1::HIS3, RAD51/rad51::HIS3*
KHSY5057	*MATα, ura3-52, trp1Δ63, hisΔ200, exo1::HIS3, rad51::HIS3*
KHSY5059	*ura3-52/ura3-52, trp1Δ63/trp1Δ63, hisΔ200/hisΔ200, EXO1/exo1::HIS3, RAD51/rad51::HIS3, SGS1/sgs1::TRP1*
KHSY5060	*ura3-52/ura3-52, trp1Δ63/trp1Δ63, hisΔ200/hisΔ200, EXO1/exo1::HIS3, RAD59/rad59::HIS3, SGS1/sgs1::TRP1*
KHSY5067	*MATa, ura3-52, trp1∆63, his3∆200, leu2∆1, lys2Bgl, hom3-10, ade2∆1, ade8, hxt13::URA3, rad59::HIS3*

KHSY5068	*MATa, ura3-52, trp1Δ63, his3Δ200, leu2Δ1, lys2Bgl, hom3-10, ade2Δ1, ade8, hxt13::URA3, rad51::HIS3*
KHSY5095	*MATa, ura3-52, trp1Δ63, his3Δ200, leu2Δ1, lys2Bgl, hom3-10, ade2Δ1, ade8, hxt13::URA3, rad51::HIS3, sgs1::TRP1*
KHSY5098	*MATa, ura3-52, trp1Δ63, his3Δ200, leu2Δ1, lys2Bgl, hom3-10, ade2Δ1, ade8, hxt13::URA3, exo1::loxP-G418-loxP, rad59::HIS3, sgs1::TRP1*
KHSY5099	*MATa, ura3-52, trp1Δ63, his3Δ200, leu2Δ1, lys2Bgl, hom3-10, ade2Δ1, ade8, hxt13::URA3, exo1::loxP-G418-loxP, rad51::HIS3*
KHSY5099	*MATa, ura3-52, trp1Δ63, his3Δ200, leu2Δ1, lys2Bgl, hom3-10, ade2Δ1, ade8, hxt13::URA3, exo1::loxP-G418-loxP, rad51::HIS3*
KHSY5102	*MATa, ura3-52, trp1Δ63, his3Δ200, leu2Δ1, lys2Bgl, hom3-10, ade2Δ1, ade8, hxt13::URA3, exo1::loxP-G418-loxP, rad51::HIS3, sgs1::TRP1*
KHSY5109	*MATa, ura3-52, trp1Δ63, his3Δ200, leu2Δ1, lys2Bgl, hom3-10, ade2Δ1, ade8, hxt13::URA3, sgs1-F1192D.TRP1*
KHSY5132	*MATa, ura3-52, trp1Δ63, his3Δ200, leu2Δ1, lys2Bgl, hom3-10, ade2Δ1, ade8, hxt13::URA3, exo1::loxP-G418-loxP, sgs1-F1192D.TRP1*
KHSY5222	*MATa, ura3-52, trp1Δ63, his3Δ200, leu2Δ1, lys2Bgl, hom3-10, ade2Δ1, ade8, hxt13::URA3, rrm3::G418, sgs1-F1192D.TRP1*
KHSY5225	*MATa, ura3-52, trp1Δ63, his3Δ200, leu2Δ1, lys2Bgl, hom3-10, ade2Δ1, ade8, hxt13::URA3, sgs1-F1192D.TRP1, srs2::G418*
KHSY5229	*ura3-52/ura3-52, leu2Δ1/ leu2Δ1, trp1Δ63/trp1Δ63, his3Δ200/his3Δ200, lys2ΔBgl/ lys2ΔBgl, hom3-10/ hom3-10, ade2Δ1/ade2Δ1, ade8/ade8, YEL069C::URA3/YEL069C::URA3, sgs1::TRP1/sgs1-F1192D.TRP1*
KHSY5233	*MATa, ura3-52, trp1Δ63, his3Δ200, leu2Δ1, lys2Bgl, hom3-10, ade2Δ1, ade8, hxt13::URA3, pol32::loxP-G418-loxP, sgs1-F1192D.TRP1*
KHSY5253	*ura3-52/ura3-52, leu2Δ1/ leu2Δ1, trp1Δ63/trp1Δ63, his3Δ200/his3Δ200, lys2ΔBgl/ lys2ΔBgl, hom3-10/ hom3-10, ade2Δ1/ade2Δ1, ade8/ade8, YEL069C::URA3/YEL069C::URA3, sgs1::TRP1/sgs1::TRP1*
KHSY5255	*ura3-52/ura3-52, leu2Δ1/ leu2Δ1, trp1Δ63/trp1Δ63, his3Δ200/his3Δ200, lys2ΔBgl/ lys2ΔBgl, hom3-10/ hom3-10, ade2Δ1/ade2Δ1, ade8/ade8, YEL069C::URA3/YEL069C::URA3, SGS1/sgs1-F1192D.TRP1*
KHSY5257	*MATa, ura3-52, trp1Δ63, his3Δ200, leu2Δ1, lys2Bgl, hom3-10, ade2Δ1, ade8, hxt13::URA3, sae2::TRP1*
KHSY5262	*ura3-52/ura3-52, leu2Δ1/ leu2Δ1, trp1Δ63/trp1Δ63, his3Δ200/his3Δ200, lys2ΔBgl/ lys2ΔBgl, hom3-10/ hom3-10, ade2Δ1/ade2Δ1, ade8/ade8, YEL069C::URA3/YEL069C::URA3*
KHSY5265	*ura3-52/ura3-52, leu2Δ1/ leu2Δ1, trp1Δ63/trp1Δ63, his3Δ200/his3Δ200, lys2ΔBgl/ lys2ΔBgl, hom3-10/ hom3-10, ade2Δ1/ade2Δ1, ade8/ade8, YEL069C::URA3/YEL069C::URA3, SGS1/sgs1::TRP1*
KHSY5274	*MATa, ura3-52, trp1Δ63, his3Δ200, leu2Δ1, lys2Bgl, hom3-10, ade2Δ1, ade8, hxt13::URA3, mre11::HIS3 , sgs1-F1192D.TRP1*
KHSY5289	*ura3-52/ura3-52, leu2Δ1/ leu2Δ1, trp1Δ63/trp1Δ63, his3Δ200/his3Δ200, lys2ΔBgl/ lys2ΔBgl, hom3-10/ hom3-10, ade2Δ1/ade2Δ1, ade8/ade8, YEL069C::URA3/YEL069C::URA3, sgs1-F1192D.TRP1/sgs1-F1192D.TRP1*
KHSY5304	*MATa, ura3-52, trp1Δ63, his3Δ200, leu2Δ1, lys2Bgl, hom3-10, ade2Δ1, ade8, hxt13::URA3, sae2::TRP1, sgs1-F1192D.TRP1*
KHSY5312	*MATa, ura3-52, trp1Δ63, his3Δ200, leu2Δ1, lys2Bgl, hom3-10, ade2Δ1, ade8, hxt13::URA3, sgs1-F1192D.TRP1, top3::G418*
KHSY5315	*MATa, ura3-52, trp1Δ63, his3Δ200, leu2Δ1, lys2Bgl, hom3-10, ade2Δ1, ade8, hxt13::URA3, exo1::loxP-G418-loxP, pol32::loxP-G418-loxP, sgs1-F1192D.TRP1*

KHSY5320	*MATa, ura3-52, trp1Δ63, his3Δ200, leu2Δ1, lys2Bgl, hom3-10, ade2Δ1, ade8, hxt13::URA3, sgs1-K706A, F1192D.TRP1*
KHSY5321	*MATa, ura3-52, trp1Δ63, his3Δ200, leu2Δ1, lys2Bgl, hom3-10, ade2Δ1, ade8, hxt13::URA3, sgs1-K706A.TRP1*
KHSY5335	*MATa, ura3-52, trp1Δ63, his3Δ200, leu2Δ1, lys2Bgl, hom3-10, ade2Δ1, ade8, hxt13::URA3, pol32::loxP-G418-loxP, rad51::HIS3*
KHSY5354	*MATa, ura3-52, trp1Δ63, his3Δ200, leu2Δ1, lys2Bgl, hom3-10, ade2Δ1, ade8, hxt13::URA3, mre11::HIS3*
KHSY5356	*MATa, ura3-52, trp1Δ63, his3Δ200, leu2Δ1, lys2Bgl, hom3-10, ade2Δ1, ade8, hxt13::URA3, sgs1::TRP1, top3::G418*
KHSY5363	*MATa, ura3-52, trp1Δ63, his3Δ200, leu2Δ1, lys2Bgl, hom3-10, ade2Δ1, ade8, hxt13::URA3, pol32::loxP-G418-loxP, sgs1-K706A, F1192D.TRP1*
KHSY5365	*MATa, ura3-52, trp1Δ63, his3Δ200, leu2Δ1, lys2Bgl, hom3-10, ade2Δ1, ade8, hxt13::URA3, pol32::loxP-G418-loxP, sgs1-K706A.TRP1*
KHSY5373	*MATa, ura3-52, trp1Δ63, his3Δ200, leu2Δ1, lys2Bgl, hom3-10, ade2Δ1, ade8, hxt13::URA3, mre11::HIS3 , sae2::TRP1*
KHSY5377	*MATa, ura3-52, trp1Δ63, his3Δ200, leu2Δ1, lys2Bgl, hom3-10, ade2Δ1, ade8, hxt13::URA3, mre11:HIS3: , sae2::TRP1, sgs1-F1192D.TRP1*
RDKY2614	*MATα, ura3-52, trp1Δ63, hisΔ200, exo1::HIS3*
RDKY2666	*MATa, ura3-52, trp1Δ63, hisΔ200*
RDKY2710	*MATa, ura3-52, trp1Δ63, hisΔ200, rad52::URA*
RDKY5290	*MATa, ura3-52, trp1Δ63, hisΔ200, sgs1::TRP1*

[a] RDKY strains are a gift from Richard Kolodner (University of California, San Diego).
