# Supplementary material for: Sgs1 Binding to Rad51 Stimulates Homology-Directed DNA Repair in Saccharomyces cerevisiae
Source: Genetics. 2017 Nov 21;208(1):125–38. doi: 10.1534/genetics.117.300545 (PMC5753853; doi:10.1534/genetics.117.300545)
Supplement: Supplementary file 2 [file 125TableS2.pdf]

Table S2. Rates of accumulating spontaneous mutations at *CAN1*, *hom3-10*, and *lys2-Bgl* loci

Genotype	**Mutation rate**					
	**Can^r**		**Hom$^+$**		**Lys$^+$**	
	(x 10^{-7})	95% CI[a]	(x 10^{-9})	95% CI	(x 10^{-9})	95% CI
wildtype	**1.5**	0.9-2	**2.2**	1.8-2.8	**4.7**	2.3-6.3
sgs1	**2.7**	1.8-4	**1.7**	1.1-2.4	**11**	7.6-12
sgs1-FD	**1.4**	1.2-1.6	**4.1**	3.3-5.3	**6.0**	4.6-11.9
pol32	**2.1**	1.1-3.4	**8.7**	5.1-12.1	**6.9**	4.5-8.9
pol32 sgs1	**1.5**	0.9-2.4	**10.0**	8.0-16.0	**10.0**	6.0-14.0
pol32 sgs1-FD	**1.3**	0.6-2	**4.3**	3.4-6.2	**3.9**	2.9-5.6

[a] 95% confidence intervals (CI) were calculated according to NAIR *et al.* 1940.
